# Quality of life versus length of life considerations in cancer patients: A systematic literature review

**DOI:** 10.1002/pon.5054

**Published:** 2019-05-15

**Authors:** Anne Shrestha, Charlene Martin, Maria Burton, Stephen Walters, Karen Collins, Lynda Wyld

**Affiliations:** ^1^ Department of Oncology and Metabolism University of Sheffield Sheffield; ^2^ Faculty of Health and Wellbeing Sheffield Hallam University Sheffield; ^3^ School of Health and Related Research University of Sheffield Sheffield

**Keywords:** cancer, decision making, longevity, quality of life, trade‐off

## Abstract

**Objective:**

Patients with cancer face difficult decisions regarding treatment and the possibility of trading quality of life (QoL) for length of life (LoL). Little information is available regarding patients' preferences and attitudes toward their cancer treatment and the personal costs they are prepared to exchange to extend their life. The aim of this review is to determine the complex trade‐offs and underpinning factors that make patients with cancer choose quality over quantity of life.

**Methods:**

A systematic review of the literature was conducted using MeSH terms: cancer, longevity or LoL, QoL, decision making, trade‐off, and health utility. Articles retrieved were published between 1942 and October 2018.

**Results:**

Out of 4393 articles, 30 were included in this review. Older age, which may be linked to declining physical status, was associated with a preference for QoL over LoL. Younger patients were more likely to undergo aggressive treatment to increase survival years. Preference for QoL and LoL was not influenced by gender, education, religion, having children, marital status, or type of cancer. Patients with better health valued LoL and inversely those with poorer physical status preferred QoL.

**Conclusion:**

Baseline QoL and future expectations of life seem to be key determinants of preference for QoL versus LoL in cancer patients. In‐depth studies are required to understand these trade‐offs and the compromises patients are willing to make regarding QoL or LoL, especially in older patients with naturally limited life expectancy.

## 1 BACKGROUND

A diagnosis of cancer can be devastating, and deciding on the appropriate treatment can be complicated and daunting. Patients are asked to consider factors that include mortality from the disease and the potential for acute and chronic morbidity from the treatment. Appropriate decision making requires satisfactory patient understanding of these treatment choices, which includes the potential benefits and harms.^1^ The primary focus of cancer treatment has always been to increase overall and disease free survival; however, quality of life (QoL) has been increasingly recognized as an important end point.^2^

Although there is an instinctive understanding of the term “quality of life,” there are multiple definitions, which gives testimony to the fact that it is a complex concept with many diverse facets and components. The standard dimensions used in QoL questionnaires measure the presence or absence of specific symptoms or overall general health. They do not measure patients' beliefs or attitudes toward treatment and intervention outcomes.^3^ Decision making in a cancer setting can be a difficult process due to its multifaceted nature. The patients' outlook and beliefs are paramount, but this is heavily influenced by their own experiences and those of friends and family.^4^ In addition, current QoL and physical status can affect subsequent decisions.

Most cancer trials primarily focus on the standard oncology end points relating to survival, but it is possible to derive composite measures, which assess the impact of QoL on the final outcome of different therapies. These are called quality adjusted survival metrics or health utility metrics, and a wide range of them have been developed over the past 30 years. Utility measures allow patients a chance to value a different perspective on treatment and outcomes. Two methods of utility measurement that may be used to calculate quality adjusted life years (QALY) or quality adjusted survival are standard gamble and time trade‐off (TTO).^5^ In standard gamble, patients are asked to choose between staying in a state of ill health for a specified time period or choosing a treatment that may either cause their death or restore perfect health. In the case of TTO, the individual expresses a preference between two choices, usually between LoL or a better health status.^4^ These methods have been increasingly adapted in cost‐utility analyses of pharmaceuticals and various health‐care interventions. In reality, scenarios are often more complex with disease and treatment effects impacting variably on QoL over a prolonged time course. There may be a significant drop in QoL after an intervention but an overall better long‐term QoL and increased life expectancy. QoL measurement should not just focus on a single time point when assessing an intervention.

In cancer treatment, patients are often required to make trade‐offs between QoL and length of life (LoL).^6^ Tumor‐specific therapy can potentially prolong life; however, this may reduce QoL significantly. Some patients are willing to endure toxicities associated with treatment in order to increase their LoL, while others value QoL more and are reluctant to spend their remaining years in a compromised state.^7^ This involves weighing the risks and benefits of treatment and managing the patients' concerns and expectations. There may be personal reasons associated with their health, the effect on their family and friends, and the consequences of the treatment itself. A trade‐off for potential gain in life expectancy may involve short‐term debility from treatment (postsurgical pain, chemotherapy‐induced nausea and alopecia, and etc) or permanent side effects (stoma, disfigurement, physical dependency, and etc). Moreover, the compromise is not always related to health but instead may be about financial burdens and increased dependency on friends and family.

To understand cancer treatment choices concerning trade‐off, various questionnaires and methodologies have been devised to understand patient preferences and priorities toward cancer treatment. Quality‐adjusted time without symptoms or toxicity (Q‐Twist) allows the combination of both quality and quantity of survival time.^8^, ^9^ The principle hypothesis of this method is that patients without disease symptoms or treatment toxicity have a better health‐related quality of life (HrQoL) than those who have disease‐specific symptoms and toxicity. Q‐TWiST was initially used to assess adjuvant therapy for breast cancer and has now been adapted in other cancers.^10^, ^11^, ^12^ The Quality/Quantity Questionnaire designed by Stiggelbout and colleagues was created to assess patients' preferences toward either QoL or LoL when deciding about cancer treatments.^7^ Other methods include discrete choice experiments and various bespoke questionnaires tailored to a specific study.^13^, ^14^, ^15^

The aim of this review was to determine the factors influencing patient preferences for either QoL or LoL and how these impacts on cancer treatment choices.

## 2 METHODS

### 2.1 Search strategy and selection criteria

A systematic literature search was performed according to PRISMA guidelines (see supporting information) using five databases between 1942 and October 2018. The databases included MEDLINE, SCOPUS, Cumulative Index to Nursing and Allied Health Literature (CINAHL), PsychINFO, and Web of Science. A pilot search on MEDLINE, was performed to identify the relevant keywords contained in the title, abstract, and subject descriptors. Five broad categories of concepts were searched: “quality of life,” “cancer,” “length of life,” “health utilities,” and “decision making.” The search terms included (cancer* OR neoplasm* OR oncolog* or tumo?r*) AND (quality of life OR QoL) AND (Longevity OR Length of Life) AND (decision making OR patient participation OR patient preference OR patient participation OR treatment choice) AND (health state utilit* OR standard gambl* OR trade‐off). See Appendix S1 for the search strategy as used in Ovid Medline. The literature search was carried out by two authors (A.S. and C.M.).

A study was only included if there was reference made to preference for QoL or LoL with or without determinants that may influence treatment choice. These factors could be either demographic influences, health status, or personal factors. Study designs could be qualitative, quantitative, or of mixed methods. Studies included were limited to adults with cancer and published in English. A PRISMA format was used to filter through articles. Editorials, reviews, and expert opinions were excluded. Hypothetical studies with healthy volunteers were also excluded as it was felt that these studies were unrealistic in their assessment of whether LoL or QoL would be favored in a cancer setting. Health status utilities were included in the search to include any trade‐off papers suitable for review. Time trade‐off studies may indicate treatment preferences, however not necessarily in the context of a preference for QoL versus LoL. Only those focusing on QoL versus LoL preferences were included.

**Figure 1:**
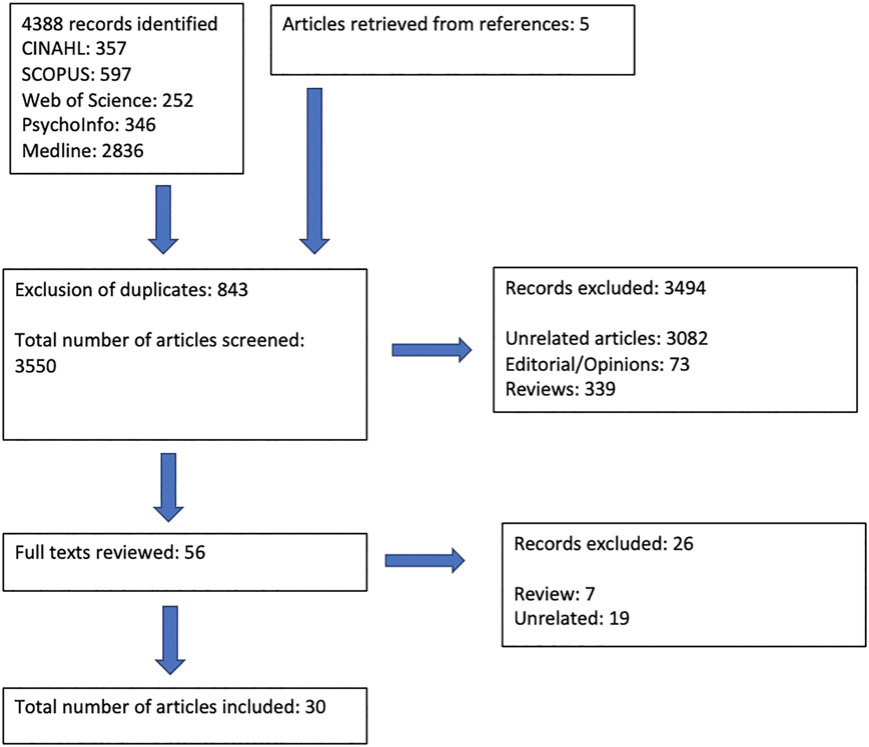
PRISMA flow chart of study selection

Study selection was by a two‐step process by two independent reviewers (A.S. and C.M.), at titles and abstract stage with arbitration for articles with uncertainty. In the second stage, full‐text articles were independently reviewed (Figure 1). Reference lists of all selected articles were reviewed to identify any additional relevant articles, identifying five further articles. When an article referred to additional publications for more details concerning study methods and design, those publications were also acquired.

### 2.2 Data abstraction

Data extraction was performed by two independent reviewers (A.S. and C.M.). The information collected included study design, aim of study, location of study, sample size and response rate, age of the sample, type of cancer, any research tools used in the form of questionnaires and the findings of the study relating to QoL versus LoL preferences.

### 2.3 Quality assessment

The Mixed Methods Appraisal Tool (MMAT) was used to quality assess the articles that were included in the study. The 2011 MMAT tool encompasses five types of mixed methods study components or primary studies: qualitative, quantitative randomized controlled trials, quantitative nonrandomized, quantitative descriptive, and mixed methods, each with its own set of methodological quality criteria. For each item the response categories were “yes,” “no,” or “can't tell” followed by comments.^16^ Higher quality is denoted by the number of stars (*) in the tables. Quality assessment was independently scored by two reviewers (A.S. and C.M.). No study was excluded based on quality assessment, as all were of acceptable quality.

## 3 RESULTS

The literature search revealed 4388 articles. A total of 843 abstracts were excluded because of duplication, and 3494 articles were declined as they were either reviews, expert opinions/editorials, or not suitable for the topic under review. A total of 56 articles were reviewed fully, and only 30 deemed suitable for inclusion. The 26 rejected papers were not suitable as they were either reviews or not relevant (Figure 1). Included studies are summarized in Tables 1 (quantitative), 2 (mixed methods), and 3 (purely qualitative) (Tables 2 and 3).

**Table 1:** Details of quantitative studies included in this review, associated with the trade‐offs related to length of life (LoL) and quality of life (QoL) (NR—not reported)

First Author and Year Published	Country	Aim	Sample Size (Response Rate %)	Mean/Median Age in Years (Range)	Type of Cancer and Stage	Questionnaires	Results Regarding QoL/LoL	Quality of Studies Using Mixed Methods Appraisal Tool (MMAT)
Kiebert (1994)^17^	Netherlands	Investigate the importance of different factors on the trade‐off Explore relationship between these importance ratings and personal characteristics	212	NR 18‐75	Testicular Breast Colorectal Lung Esophagus Lymphoma Skin Prostate	Self‐designed questionnaire	A priori chance of survival and baseline QoL considered important factors in choice of LoL or QoL	**
Stiggelbout (1996)^7^	Netherlands	Assess QoL versus LoL	211 NR	NR <30‐<71	Breast Testicular Colorectal lung	• QQ Questionnaire • Medical outcome short form general health survey MOS SF‐20) • Rotterdam symptom checklist (RSCL)	• Younger patients preferred LoL • Those with poorer physical function preferred LoL than QoL • No difference in patients with cancer with good prognosis, ie, breast/testicular versus recurrent colorectal/lung	***
Helgason (1996)^18^	Sweden	Identify and measure the important disease‐specific distress for patients with prostate cancer	319 73	NR 50‐80	Prostate cancer	Radiumhemmets Scale of Sexual Function	63% of patients stated they would trade off the possibility of longer life over intact sexual function.	****
Perez (1997)^3^	New Zealand	Assess how patients perceive their illness and make decisions about treatment.	124 62	66 18‐91	Metastatic cancer of any type	Spitzer Quality of life Index and Uniscale	• 37% were prepared to trade time for better QoL, 39% too well to consider any trade‐off, 24% did not want to trade time. • Patients willing to trade time had lower score in four out of domains	***
Weeks (1998)^19^	United States	Do terminally ill patients understand their prognosis and treatment preference associated with comfort over life extension	917 55	62 NR	Stage III/IV lung cancer metastatic colon cancer	• Activities of daily living • Interview	Patient who thought their life expectancy was greater than 6 mo wanted life prolonging treatment	**
Silvestri (1998)^20^	United States	Assess treatment preferences by those who completed chemotherapy for nonsmall cell lung cancer and minimum survival benefit	81 100	<60‐>70	Stage III and IV non‐small cell lung cancer	Scenario based	6% would have chemotherapy for even 1 wk of extra survival, 11% would not have chemotherapy even if there was potentially 24 moof increased survival.	***
List (2000)^21^	United States	Determine patients' pretreatment choice regarding treatment effects and survival	131 96	59 29‐87	Head and Neck Stage II to IV	• FACT H + N • Performance status scale for head and neck (PSS‐HN) • Karnofsky Performance Scale • Bespoke 12‐item prioritization scale	75% ranked being cured of cancer as being most important, 56% felt living as long as possible as an important priority. Those with better QoL wanted to be cured of cancer.	***
Perez (2001)^22^	New Zealand	Measure the application of time trade‐off utility measure	64 84	58.7 30‐80	Advanced breast cancer	Spitzer QoL Index and Uniscale	63% wanted to trade time, 32% felt they were too well to trade time.	****
Donovan (2002)^23^	United States	Assess women's preferences for treatment in the case of recurrent ovarian cancer and identify factors associated with treatment preference	81 NR	60.0 NR	Recurrent Ovarian Cancer	• Profile of Mood States—Short Form • The Systems of Belief Inventory—15R • Satisfaction with Life Scale • Functional Assessment of Chronic Illness therapy—Spiritual Well‐Being Scale (FACIT‐Sp) • FACT‐G • FACT‐O • Decision Board Exercise	• Women with ovarian cancer preferred salvage therapy to palliative treatment, in hope to increase LoL. QoL was a secondary consideration. • Iinitial treatment preference was not related to age, marital status, number of children, or employment status.	***
Koedoot (2003)^24^	Netherlands	To what extent does information from friends, family, and doctors affect treatment choice	140 68	NR 26‐82	Various types of metastatic cancer	• Karnofsky Index • Rotterdam Symptom Checklist • Cancer Locus of Control Scale • Michigan assessment of decision style • QQ Questionnaire	• 81% proposed that doctor suggested chemotherapy • Younger patients had a stronger preference for chemotherapy • Patients striving for QoL did not want chemotherapy	****
Meropol (2003)^25^	United States	Understand the difference in perception and decision‐making regarding participation in phase 1 cancer treatment trial in patients and doctors	328 55	>18 NR	Advanced Cancer – not specified (31 different types)	• Control preference scale • Decisional conflict scale • SF‐12 • EuroQoL Health State Thermometer • Self‐designed questionnaire	5% of subjects responded LoL was more important	***
List (2003)^26^	United States	Examine and compare the treatment priorities of newly diagnosed advanced stage head and neck cancer with a control group.	247 NR	58 25‐87	Head and neck II – IV	• FACT‐HN • PSS‐HN • 12 item priority scale	• Married prioritized LoL • Younger patients valued LoL more important than older patients.	***
Derks (2005)^27^	Netherlands	Assess how age, sociodemographic data, comorbidity, social support depressive symptoms, and QoL influence treatment choice.	266 NR	NR 45‐>80	Head and neck Stage II‐IV	• EORTC‐QLQ‐C30 • EORTC‐QLQ‐H&N35 • Centre for • Epidemiological studies Depressive Scale (CES‐D) • Social Support List‐Interactions (RSS12‐I) • QQ questionnaire Questionnaire	• 89% in 45 to 60 age group received standard treatment compared with 62% in greater than 70 years old. • Elderly patients receiving non‐standard treatment reported QoL compared with those receiving standard treatment.	***
Jansen (2006)^28^	Netherlands	Determine quantitatively patients' perceptions of choice regarding treatment with adjuvant chemotherapy	719 62	NR 32‐89	Breast Cancer	Self‐designed questionnaire	Greater than 80% patients underwent chemotherapy as LoL was considered important	****
Meropol (2008)^13^	United States	Understand how patient preference (QoL/LoL) impact decision making	748 68	>18 NR	Advanced cancer—not specified	• Short‐Form (SF‐12) • Revised Impact of Events Scale (RIES) • QoL and LoL preference	• 65% of patients felt QoL was more important than LoL; however, LoL matters, 19% thought vice versa, 15% thought QoL is all that matters, and 1% thought LoL was all that mattered. • Overall 55% felt both were equally important.	***
Wong (2013)^29^	United States	Assess patient characteristics that influence trade‐offs	584 68	61 27‐90	Breast, prostate, GI, lung, head/neck, skin, hematological, other	Discrete choice questionnaire	Patients with higher income favored LoL.	****
Laryionava (2014)^6^	Germany	Validate QQQ in the German Population	309 77	52 16‐88	• Breast • Lung • Kidney • Prostate • Colon • Rectum • Pancreatic • Bladder • Others	• QQ Questionnaire—Functional Assessment of Cancer Therapy—General (FACT‐G) • Cancer Communication Assessment Tool for patients (CCAT‐P) • Questionnaire on Stress in Cancer Patients (QSC‐R10) • Positive and Negative Quality in Marriage Scale (PANQIMS)	• No difference in QoL and LoL in age, gender, patients with children, and education • Unemployed patients preferred QoL to LoL • Family involvement in decision making correlated to LoL	***
Marta (2014)^14^	Brazil	Assess the choices and priorities of patients with cancer, health care professionals, and lay person regarding quantity and QoL	250 85.6	56 NR	Gastrointestinal, breast, heamatological, lung, other	Self‐designed questionnaire	21% of the patients agreed they would opt for treatment that prolongs survival, regardless of QoL; 15% would opt for treatment that would optimize QoL.	***
Krammer (2014)^30^	Germany	Examine attitudes toward melanoma therapy options and QoL versus LoL	30 NR	57.5 25‐87	Melanoma	Bespoke Questionnaire	• 44% of the patients were prepared to accept side effects for longer survival. One‐third of the patients would rather live 1 mo longer than have a higher QoL at the end of their life. • Older patients less likely to undergo treatment.	****
Malhotra (2016)^31^	Singapore	Compare the attitudes of QoL and LoL between community dwelling older adults (CDOA) and advanced cancer patients	1387 NR	62 NR	Stage 4 cancer (all)	QQ Questionnaire	Overall QoL valued more than LoL. Cancer patients valued LoL above than QoL compared with the CDOA.	***
Danson (2016)^32^	United Kingdom	Assess health‐related quality of life (HRQoL) and smoking status at diagnosis and preference for treatments, which promote QoL over LoL depending on smoking status	304 47.4	65.6 51‐80	Advanced Lung cancer	• EORTC‐QLQ‐C30 • EORTC‐QLQ‐LC13 • QQ questionnaire	Significant preference for QoL over LoL irrespective of smoking status	****
Pisu (2017)^33^	United States	Examine concerns of ovarian cancer patients and whether it varies in 2different age ranges	170 66	61.8 24‐90	Ovarian Stage 1 to 4	Self‐designed Questionnaire	Patients felt maintaining QoL and living as long as possible both very important regardless of age.	****

**Table 2:** Details of mixed method studies included in this review, associated with the trade‐offs related to length of life (LoL) and quality of life (QoL) (NR—not reported)

First Author and Year Published	Country	Aim	Sample Size and (Response Rate %)	Mean/Median Age in Years (Range)	Type of Cancer and Stage	Questionnaires	Results Regarding Qol/LoL	Quality of Studies Using MMAT
Sekeres (2004)^34^	United States	Explore factors influencing the choice of induction chemotherapy or supportive care	43 98	71 60‐85	Acute myeloid leukemia Advanced myelodysplastic syndrome	• FACT‐G • FACT‐An (Anemia) • SF‐12 • Interview	97% agreed QoL was more important than LoL	***
Voogt (2004)^35^	Netherlands	Assess patients' attitudes toward medical treatment	200 66	63.5 NR	• Breast • Colorectal • Ovarian • Prostate • (All advanced cancer)	• QQ Questionnaire • Positive and Negative affect scale • EORTC‐QLQ‐C30 • Interview	• Younger patients preferred LoL • Patients without partner preferred QoL • No difference in sex, children, education, religion, and type of cancer. • Short history of cancer preferred LoL, patients preferred QoL were closer to death • attitudes did not change at 6 and 12 mo	**
Jenkins (2013)^36^	United Kingdom	Examine the experience and preferences of patients with advanced ovarian cancer regarding care and treatment	225 52	63.5 31‐83	Ovarian cancer 1 to 4	• EORTC QLQ C30 • EORTC QLQ INFO25 • Interview	33% prioritized QoL as important, 9% prioritized LoL, and 57% felt both were important.	***
Collins (2013)^37^	United States	Identify common themes from patient responses and identify factors associated with whether they would undergo palliative intervention in advanced cancer to relieve symptoms	98 NR	59 23‐86	NR but patients were admitted with bowel obstruction/perforation, gastrointestinal bleed, abdominal pain, obstructive jaundice, malnutrition, and infection.	• FACT‐G • Interview	• 20 patients would undergo palliative intervention to treat cancer or live longer. • 47% for symptom control/better QoL • Physicians' recommendation was a strong influence	***
DiBonaventura (2014)^15^	United States	Understand how patients' trade off medication side effects with effectiveness and/or improved QoL	181 7	52.2 NR	Metastatic Breast cancer	• FACT‐B • FACT‐G • Interview	Treatment effectiveness (overall survival) most important to choosing chemotherapy for metastatic breast cancer	***

**Table 3:** Details of purely qualitative studies included in this review, associated with the trade‐offs related to LoL and QoL (NR – not reported)

First Author and Year Published	Country	Aim	Sample Size and (Response Rate %)	Mean/Median Age in Years (Range)	Type of Cancer and Stage	Questionnaires	Results Regarding Qol/LoL	Qualitiy of Studies Using MMAT
Gerber (2012)^38^	United States	To gain insight into patients' perceptions of maintenance chemotherapy	13 27	62 39‐69	Lung cancer	Focus group interview	Trade‐off issues highlighted “… with the maintenance are we going to be able to go on with life, so not just be totally ill all the time or do we want to take a chance and be with our family and loved ones and have some quality of life left?”	***
Brom (2014)^39^	Netherlands	Obtain insight into patients' preferences and the reasons for patients' ideas of preferred role in treatment decision making whether to start a life prolonging treatment	28 (NR)	NR 18‐>81	• Glioblastoma • Metastatic colorectal cancer	Interview	• Some patients felt they would stop treatment if it affected QoL. • Several patients felt “doing nothing” wasn't an option and unwilling to accept transition from LoL to QoL to death.	***
Berry (2015)^40^	United States	Explore and understand the aspects and process of treatment decision making perceived by patients with bladder cancer	60 42	66 33‐86	Bladder cancer stage 0a‐4	Interview	38% felt survival was the main feature of treatment decision, balancing toxicities and LoL.	***

The majority of studies identified in this review were quantitative. Generic questionnaires (EORTC‐QLQ‐C30 and FACT‐G) and disease specific questionnaires (EORTC‐QLQ‐H&N) were used to assess QoL. The studies were mainly conducted to understand the decision‐making process in the advanced cancer setting. The studies had wide focus that included understanding the role of the doctor and the attitude the patient has toward their treatment, among other themes. Understanding QoL and LoL trade‐offs as part of the decision‐making process, usually formed a limited part of many of these studies.

### 3.1 QoL versus LoL

Meropol and colleagues (2008) suggested that QoL and LoL are both equally important; however, the majority of patients with advanced cancer in this study prioritized QoL over LoL.^41^ This was also reflected by the study of Jenkins and associates.^36^ Silvestri and associates noted although there were some patients who would endure treatment and associated toxicities just to live a single day longer, there were also patients who would decline all treatments. These latter patients would rather maintain their QoL and having to withstand the adverse effects of treatment would not be a worthwhile trade‐off.^20^ The authors postulated that patients may opt for enhanced QoL only if the chance of survival was less than 50% relative to baseline survival (without treatment).^42^

Many patients in the study by Brom and colleagues felt that they ought to have some sort of intervention for their cancer and found it difficult to accept the concept of LoL and QoL. Although some patients opted for treatment initially, they expressed the view that if it was affecting their QoL, they would cease treatment.^39^ Marta and colleagues noted that the majority of patients in their study wanted to undergo a treatment that would prolong life but not compromise their QoL.^43^ In a qualitative study by Gerber and colleagues, patients stated that they were keen to maintain their activities and not be a burden on family, and therefore not undergo chemotherapy if those factors were compromised, indicating the importance of QoL.^38^

### 3.2 Survival and baseline QoL

Survival seemed to be a key feature in the decision‐making process and patients were found to opt for treatment if they felt that their prognosis was likely to improve.^15^, ^19^, ^28^, ^40^ Their current health status also affected their choice. Perez and associates found that those who wanted to trade time, scored lower in many of the domains of the baseline HRQoL questionnaires.^3^ Patients in better health were found to rate LoL more highly, whereas those who were in poorer health strived to maintain their QoL.^7^, ^22^, ^32^, ^44^ Kiebert and associates noted that issues patients felt were important were baseline QoL and the probability of survival.^17^

### 3.3 Demographic factors

Kiebert and associates assessed factors affecting decision making for cancer treatment and noted that important factors were age, marital status, children, inability to work due to side effects, disease related life expectancy, and baseline QoL. No significant associations were found between the various determinants; however, patients did rate having children and marital status as somewhat important in decision making.^17^

Other studies have shown different results, with gender, children, education, religion, and cancer type not influencing treatment choices.^3^, ^6^, ^23^, ^35^ Those with strong family links preferred survival. Unemployed patients prioritized QoL.^6^ Wong and colleagues concluded that those who were able to pay for their treatment chose to have treatment to prolong their life.^45^ These latter findings are only relevant in self paying health care systems.

Many of the studies carried out have not been age specific; therefore, it has been difficult to make inferences about the influence of age on LoL/QoL preferences. The studies in this review show a mixed picture. Older patients have a preference for QoL, which is not surprising considering natural limitations to life expectancy and the often reduced QoL associated with advanced age.^34^ Younger cancer patients were more likely to tolerate aggressive treatments to increase survival years.^30^, ^35^, ^46^ A study by Pisu and colleagues involving 170 ovarian cancer patients, showed that maintaining QoL and living as long as possible were both important. In women less than 65 years old, 96.9% felt longevity was important, and 95.9% felt that preserving QoL was important, compared with 87.5% and 90.3%, respectively, in the greater than 65‐year‐old age group.^33^ Stiggelbout and associates noted that when age was adjusted for in their statistical calculations, those in relationships and with children preferred longevity.^7^ Derks and colleagues found that older patients were less likely to receive standard treatment, an effect that was more evident in those above the age of 80 years old. Reasons behind this included lack of social support and being widowed. Patients who did not receive standard treatment also prioritized QoL more strongly.^27^

### 3.4 Symptom trade‐off

When looking at symptom tradeoffs against longevity, patients were prepared to tolerate certain treatment side effects to live longer. Patients were willing to prioritize survival over intact sexual function in prostate cancer for instance.^18^, ^44^ When patients with advanced cancer reached the end of their lives and had to endure pain and discomfort, 47% of patients chose to have palliative surgery to maintain or enhance their current health status and independence.^37^

### 3.5 Cancer‐specific trade‐off

Patients suffering from cancers with a good prognosis such as breast and testicular cancers, compared with recurrent colorectal or lung cancer had similar thoughts regarding QoL and LoL.^7^ Despite the type of cancer, patients felt that QoL and LoL were equally important when considering treatment.^41^ In the study by Pisu and colleagues involving ovarian cancer, more than 90% stated that QoL and LoL were equally important.^33^ Another study by Jenkins and associates, involving participants with ovarian cancer showed that 57% felt LoL and QoL were equally important, 9% prioritized LoL, and 33% favored QoL.^36^ However, Donovan and colleagues demonstrated that women who had recurrent ovarian cancer, would opt for LoL, and choose to receive aggressive treatment, QoL was a secondary issue.^23^ Patients with a shorter history of cancer preferred LoL; however, those with poorer prognosis and closer to their predicted time of death valued QoL more.^35^ In contrast, Meropol and colleagues found that there was no association between time since diagnosis and QoL/LoL preference.^41^

## 4 DISCUSSION

This study presents the first comprehensive review of studies looking at trade‐offs between QoL and LoL in a cancer setting. The aim of this review was to highlight whether patients prioritize QoL or Lol and the determining factors that influence the decision‐making process for cancer treatment. In fact, the findings indicate that many of the studies do not directly test determinants. The QQ questionnaire has been designed specifically to quantify the patient's choice of QoL or LoL and also to what extent patients would be inclined toward either. The questionnaire does not capture the psychological reasoning behind the preference however. It is also perhaps more suited for patients with advanced cancers where the cancer will inevitably cause death regardless of whether it was treated or not.^7^ For some patients, where curative treatments may be available, albeit with a high cost (for example, mutilating operations leading to disfigurement, ie, head and neck resections, mastectomy, and amputations) or where death due to old age or other, noncancer comorbidities is imminent; this trade‐off may also be relevant and the QQ tool is not designed to explore these scenarios.

This review highlights the importance of carrying out baseline QoL assessments prior to treatment and evaluating the impact of life expectancy. The importance of performing age specific studies is also noted as priorities between younger and older patients are different. The preferences for QoL or LoL by younger patients, may be influenced by their desire to spend time with their partner or children. Older patients are more likely to suffer from multiple comorbidities and be frailer, and discussions may need to include whether a treatment will be tolerated less well because of these limitations, or result in an increased risk of harm. Considerations should include patient intolerance to certain chemotherapy agents or surgery, as well as an understanding that they may never reach their preoperative baseline physical fitness again after treatment. This “step down” in function tends to be more prominent in the older age group,^47^, ^48^ an effect that is widely recognized across many medical interventions in older patients. They may feel that time spent receiving treatment may not be worth the extension of life for a relatively short period. Older individuals have a good overall understanding that they have lived their lives and are more accepting of the inevitability of death and of their physical limitations. Studies suggest that a good QoL in older people is often based around the following: independence, a strong social circle, and an ability to retain their “inner selves.”^49^ These values may be compromised by having treatment. Other studies have shown that the most consistent factor influencing treatment decision making in older patients is a recommendation from doctors.^50^ In breast cancer, undertreatment is well‐documented in older patients.^51^ This has led to avoidable disease‐specific deaths.^52^ Exploring the patients' views regarding treatment at an early stage would help reduce the impact of age‐related clinician bias, which is well recognized.^53^

## 5 CONCLUSIONS

Decision making in cancer treatment is difficult as there are multiple components to consider aside from the purely medical aspects. Likewise, the compromises the patient is willing to make can vary greatly depending on many factors including patient age, personal family dynamics, social structures, and, patients' likely survival and baseline QoL. This may subsequently impact on whether the patient is more inclined towards longevity or QoL. Although there are studies trying to understand the factors influencing the final decision, there is limited information on preferences between QoL and LoL and the trade‐off the patient is willing to make. Clinicians have influence over the final decision, and therefore it is vital for the patient to have a full understanding of their treatment and the impact it may have on their life.

### 5.1 Study limitations

This study is the first to use a rigorous and systematic approach to review studies based on patient preferences regarding QoL or LoL in a cancer treatment setting. Despite a comprehensive database search strategy, it is possible that some relevant articles may have been missed and despite the various methodologies, all papers included were of an acceptable design and standard for inclusion. However, the main findings of the review are likely to be robust to missing studies. On the basis of our interpretation and weighting of the evidence, we are confident in the conclusions that have been drawn from findings across several studies rather than be based on isolated studies. None of the studies in this review has looked at the impact of preexisting, noncancer‐related limitations to life expectancy as part of this trade‐off, such as is seen in the oldest age groups and the impact of acceptance of impending age‐related mortality. With the aging of Western populations, this is an important gap in the literature.

The studies included in this review are exploratory cohort studies carried out in a retrospective manner, whereby patients have already made their decision regarding treatment. There may be a source of bias influencing their responses, as many issues may not have been considered prior to treatment or the decision‐making process.

Many of these studies have mainly focused on advanced cancers of all types. For patients who are facing mortality imminently, the decision to prioritize QoL and LoL is pertinent. In the case of slow growing cancers such as prostate and breast cancers, where conservative management is widely accepted, the choice between QoL and LoL can be more complicated. Patients often die from other causes rather than the cancer itself.^54^ As the majority of the articles identified in this search did not involve early stage cancer, it is difficult to know what patients envisage from their treatment and what trade‐offs they were willing to make as well as how these factors may change with the course of the natural disease process. This is where patients' age and comorbidities may play a larger role in whether the patient opts for QoL or LoL.

### 5.2 Clinical implications

This review has several important clinical and research implications. With treatment and care now becoming more patient centered, it has become more pertinent to understand the impact of the cancer diagnosis on the patient and the motivations behind their treatment choices. The impact of treatment of certain cancers may be extreme and may involve a great deal of compromise and acceptance of change in circumstances. Factoring the likely impact of treatments on QoL relative to that at baseline should be discussed with every patient. This would ensure that patients have a full understanding of what their treatment entails and that they are aware of the consequences of treatment and nontreatment. Further in‐depth studies are required to understand the emotional and physical considerations and personal priorities the patients may have during the decision‐making process. This may go a long way in elucidating what aspects of their life they are willing to trade to maintain their QoL or increase LoL. Older age specific issues and cancer specific decision‐making processes also need exploring.

## CONFLICTS OF INTEREST

The authors have declared no conflicts of interest. The views expressed are those of the authors and not those of the NHS, the NIHR, or the Department of Health.

## Supporting information




**Data S1.** PRISMA checklist.

**Appendix S1.** Search Strategy used in Ovid (Medline).
