# Supplementary material for: Quality of life versus length of life considerations in cancer patients: A systematic literature review
Source: Psychooncology. 2019 May 15;28(7):1367–80. doi: 10.1002/pon.5054 (PMC6619389; doi:10.1002/pon.5054)
Supplement: Supplementary file 2 — Appendix S1. Search Strategy used in Ovid (Medline). [file PON-28-1367-s002.docx]

Appendix S1

Search Strategy used in Ovid (Medline)

1. Cancer*
2. Neoplasm*
3. Oncolog*
4. Tumo?r*
5. 1 OR 2 OR 3 OR 4
6. Quality of life
7. QOL
8. 6 OR 7
9. Length of life
10. Longevity
11. 9 OR 10
12. Decision making
13. Patient participation
14. Patient preference
15. Treatment choice
16. 12 OR 13 OR 14 OR 15
17. Trade off
18. Health utilit*
19. Standard gambl*
20. 17 OR 18 OR 19
21. 5 AND 8 AND 11 AND 16 AND 20
22. 5 AND 8 AND 20 AND 16
